# Diagnostic Accuracy and Confidence of [18F] FDG PET/MRI in comparison with PET or MRI alone in Head and Neck Cancer

**DOI:** 10.1038/s41598-020-66506-8

**Published:** 2020-06-11

**Authors:** Jisang Park, Kyoungjune Pak, Tae Jin Yun, Eun Kyoung Lee, Inseon Ryoo, Ji Ye Lee, Inpyeong Hwang, Roh-Eul Yoo, Koung Mi Kang, Seung Hong Choi, Chul-Ho Sohn, Gi Jeong Cheon, Ji-hoon Kim

**Affiliations:** 1 Department of Radiology, Konkuk University Chungju Hospital, 82, Gukwondae-ro, Chunju, Chungcheongbuk-do 27376 Republic of Korea; 2 Department of Nuclear medicine, Pusan National University Hospital, 179, Guduk-ro, seo-gu, Pusan 49241 Republic of Korea; 3 Department of Radiology, Seoul National University Hospital, 101, Daehak-ro, Jongno-gu, Seoul 03080 Republic of Korea; 4 Department of Radiology, Seoul National University Hospital Healthcare System Gangnam Center, Gangnam Finance Center 152, Teheran-ro, Gangnam-gu, Seoul 06236 Republic of Korea; 5 Department of Radiology, Korea University Guro Hospital, 148, Gurodong-ro, Guro-gu, Seoul 08308 Republic of Korea; 6 Department of Nuclear medicine, Seoul National University Hospital, 101, Daehak-ro, Jongno-gu, Seoul 03080 Republic of Korea

**Keywords:** Head and neck cancer, Cancer imaging, Oral cancer detection

## Abstract

The usefulness of PET/MRI in head and neck malignancy has not been fully elucidated. The purpose of our study was to evaluate the diagnostic accuracy and confidence of PET/MRI in comparison with PET or MRI alone. This study included 73 consecutive patients who underwent [18F] FDG PET/MRI in head and neck under the suspicion of malignancy. A neuroradiologist and a nuclear medicine specialist reviewed MRI and PET images, respectively and independently, followed by a consensus review of PET/MRI one month later. For 134 lesions, accuracy and confidence were compared among PET, MRI, and PET/MRI. For lesion base, PET/MRI had a sensitivity of 85.7%, a specificity of 89.1%, a PPV of 89.6%, a negative predictive value of 85.1%, and an accuracy of 87.3%. AUCs of PET/MRI per lesion (0.926) and per patient (0.934) for diagnosing malignancy were higher than PET (0.847 and 0.747, respectively) or MRI (0.836 and 0.798, respectively) alone (P < 0.05). More than 80% of the cases (111/134) showed diagnostic concordance between PET and MRI. PPV of PET/MRI was higher in malignant concordant cases (93.2%, 55/59) than in discordant cases (62.5%, 5/8) (p = 0.040). Confident scoring rate in malignant concordant cases was higher on PET/MRI (96.6%, 57/59) than on MRI (76.3%, 45/59) (p = 0.003). In conclusion, compared with PET or MRI alone, PET/MRI presents better diagnostic performance in accuracy and confidence for diagnosis of malignancy. PET/MRI is useful in patients with head and neck cancer.

## Introduction

Head and neck cancer ranks within the ten most common cancers in the human body^1,2^. Head and neck is an anatomically complex region which performs various physiological functions and constitutes the facial morphology. As a result, recurrent or residual cancer in this region tends to be challenging to treat^2^. Therefore, for patients with head and neck cancer, accurate localization of primary and recurrent tumor is essential for planning optimal treatment to preserve its function and morphology as much as possible.

There are definite advantages in using 18F-FDG PET to reveal metabolic information in the whole-body with just one examination. However, poor spatial resolution of PET is often not suitable to evaluate the lesions within the complex anatomy of the head and neck, and the physiologic uptake of FDG in this region may hinder an accurate diagnosis of PET^3^. In this regard, PET/CT has been well known to complement PET and CT, respectively^4,5^. It has come to be known as an established diagnostic modality for the evaluation of patients with head and neck cancer^6^.

Meanwhile, along with CT, MRI has been used as an important diagnostic tool for evaluation of head and neck cancer^6^. As Boss *et al*. presented the feasibility of whole-body PET/MRI systems to combine the unique metabolic imaging capabilities of PET with anatomical merits of MRI in head and neck region^7^, PET/MRI appears to be a promising modality for patients with head and neck malignancies as well as patients with suspected dementia^8^. There have been a few reports that suggested the feasibility and the potential of PET/MRI in the head and neck region^9–15^. However, until now, the usefulness of PET/MRI in the evaluation of head and neck malignancies has not been fully elucidated.

In this regard, the purpose of our study was to evaluate the diagnostic performance of PET/MRI in comparison with PET or MRI alone in diagnosing malignancy in the head and neck region.

## Materials and Methods

This retrospective study was approved by the Institutional Review Board of Seoul National University Hospital, and informed consent was waived by the Institutional Review Board of Seoul National University Hospital. We confirm that all methods in our study were performed in accordance with the relevant guidelines and regulations.

### Patient selection

We reviewed the radiology database of Seoul National University Hospital between December 2012 and August 2013, and identified 108 consecutive patients (age range, 18–83 years; mean age, 58 years; 37 women, 71 men) who underwent simultaneous PET/MRI in head and neck for work-up of malignancy. The periods of data enrolment were chosen to avoid periods when PET/MRI were clinically exposed to at least one of two reviewers (T. J. Y., 11-year experience in head and neck radiology; K. J. P., 8-year experience in nuclear medicine).

We included patients with head and neck benign or malignant lesions that were confirmed by the following diagnostic references, which are, nodal lesions, where only pathologic results were adopted for both benign and malignant diagnoses. For non-nodal lesions, malignant diagnosis adopted only pathologic results but benign diagnosis adopted both pathologic results and clinical assessment based on follow-up for longer than 6 months. The following patients were excluded; those who did not have diagnostic reference (n = 27), those who underwent PET/MRI after the biopsy (n = 04), those who underwent intervening treatment in the period between PET/MRI and biopsy (n = 04). Finally, 73 patients (age range, 47–83 years; mean age, 59 years; 25 women, 48 men) were enrolled for the analysis.

### Baseline characteristics

Table 1 summarizes clinical characteristics of the patients. In more than 70% of the cases, the primary foci of the tumor were aerodigestive tract (57/73) and the pathology was squamous cell carcinoma (54/73). In half of the cases, the indication of PET/MRI was initial work-up and work-up of recurrences, respectively.

**Table 1:** Patient Characteristics (Baseline characteristics).

Characteristics	Number
**Primary tumor sites**	
Pharynx	35 (47.9%)
Oral cavity	20 (27.4%)
Sinonasal cavity	7 (9.6%)
Parotid gland	4 (5.5%)
Larynx	2 (2.7%)
Infratemporal fossa	2 (2.7%)
Others*	3 (4.1%)
**Primary tumor pathology**	
Squamous cell carcinoma	54 (74.0%)
Poorly differentiated carcinoma	5 (6.8%)
Lymphoma	4 (5.5%)
Adenocarcinoma	3 (4.1%)
Others**	7 (9.6%)
**Study indication**	
Initial work-up of malignancy	40 (54.8%)
Work-up for recurrence	33 (45.2%)

^*^One in each of the followings: skin, conjunctiva, and external auditory canal.

**One in each of the followings: Warthin’s tumor, mucoepidermoid carcinoma, pleomorphic adenoma, carcinoma ex pleomorphic adenoma, lymphoepithelial carcinoma, liposarcoma, and chondrosarcoma

### Imaging protocol

All acquisitions were performed in the integrated simultaneous PET/MRI system (Biograph mMR, Siemens Healthcare, Erlangen, Germany). PET/MRI system consists of 3.0 Tesla MR and a fully integrated PET detector with a 16-channel head and neck surface coil, four 6-channel body coils, and eight 3-channel spine coils. All patients fasted for 6 hours before the scan. Serum glucose levels were tested before the injection and were less than 200 mg/dl in all patients. At 50 minutes after injection of 0.14 mCi/kg of ^18^F-FDG, the patient was placed on the PET/MRI scanner bed.

After a scout image was obtained by a simple fast sequence of MRI, a whole-body PET scan was performed that covered from the head to the distal thigh. Each bed PET was performed with an acquisition time of 3 min, 25.7 cm bed length with 30% of overlapping area, 4.1  ×  2.6  ×  3.1 mm and 172 matrices. Simultaneously, MR image acquisition was performed per bed. Automatic attenuation correction of PET data was done according to attenuation maps generated by the two-point Dixon sequence. After whole body PET/Dixon-VIBE PET/MRI was obtained, dedicated head and neck MR imaging was performed with simultaneous regional PET scanning. Regional PET was performed with an acquisition time of 10 min, a voxel size of 1.6  ×  1.6  ×  1.6 mm and 344 matrices.

The MR images included multiplanar spin-echo T1-weighted images (TR/TE/NEX, 570/9.7/1-2), multiplanar fast spin-echo T2-weighted images (TR/TEeff/NEX, 6885/72/1–2) with or without fat saturation, and multiplanar spin-echo T1-weighted images with or without fat saturation after the intravenous injection of 0.1 mmol/kg gadoterate meglumine (Dotarem, Guerbet, France). All images were obtained with a 3–6 mm section thickness, 1–2 mm intersection gap, 256–512 × 128–256 matrix, and 18–26 × 18–26 cm FOV

### Interpretation of images and diagnostic reference

A neuroradiologist (T.J.Y.) and a nuclear medicine specialist (K.J.P.) independently reviewed MR images and PET images, respectively, under the suspicion of tumor or tumor recurrence.

Clinical data were provided to the two reviewers by a neuroradiologist (J. S. P. with 8 years’ experience), as would be available for clinical readings, but without notifying any results of other concurrent imaging studies if performed.

The images were reviewed to detect the lesions, with special focus on the location of the clinical problem and the location where should be evaluated in patients with suspected primary or recurrent malignancy. They tried to find any other additional MRI or PET abnormality as well.

Each lesion was scored on a five-point scale for the probability of malignancy. A score of 1 indicated definitely benign; 2, probably benign; 3, equivocal; 4, probably malignant, and 5, definitely malignant.

To find and score the lesions, MR images were interpreted on the basis of known malignant imaging features with respect to size, necrosis, signal intensity, enhancement, marginal irregularity, and invasion to the adjacent structures^16–21^.

As for PET, possibility of physiologic uptake and maximum standardized uptake value (SUV max) of FDG were mainly considered to detect and score the lesions. In general, the lesions of physiologic FDG uptake were considered as benign with SUV < 2.5 (score of 1, definitely benign) or SUV ≥ 2.5 (score of 2, probably benign), while those with abnormal FDG uptake were considered as malignant with 2.5 ≤ SUV < 5 (score of 4, probably malignant) or SUV ≥ 5 (score of 5, definitely malignant). The lesions which could not be categorized as either benign or malignant were considered as equivocal (score of 3)^4,22–24^.

One month after the independent reviews of MRI and PET, the two reviewers together, reviewed the PET/MR images to reach the final consensus for the lesions, which was again moderated by a neuroradiologist (J.S.P.).

Among all the lesions that were graded by any reviewer, the lesions clarified by the diagnostic references were enrolled for analysis. The lesions which had pathologic results, but were not graded by any reviewer were also included for the analysis. The lesions that were not graded by any reviewer on MRI, PET, or PET/MRI were assigned a score of 1 (definitely benign).

Finally, a total 134 lesions were enrolled for the analysis, based on the confirmation by pathology (n = 111) and clinical follow-up (n = 23) during mean 28 months (range, 8–84 months). They were categorized into the lesions in initial work-up (n = 79) and the lesions in work-up for recurrence (n = 55). They were 72 non-nodal lesions (benign lesions [n = 32; clinical, n = 23; pathological, n = 9] vs. malignant lesions [n = 40, all pathological]) and 62 nodal lesions (benign lesion [n = 46, all pathological] vs. malignant lesion [n = 16, all pathological])

Outside of the head and neck region, distant metastases were found in 3 patients, at lung, spine, and abdominal organs and lymph nodes, respectively. These findings were not included for analysis.

### Statistical analysis

Sensitivity, specificity, positive predictive value (PPV), negative predictive value (NPV), and accuracy were obtained, where required.

For two-tiered classification of the scores between benign and malignant diagnoses from imaging interpretation, the score 1–3 were considered as a benign diagnosis and the score 4–5 were considered as a malignant diagnosis.

In addition, we compared the diagnostic performance among the three modalities (MRI vs PET vs PET/MRI), per person base and lesion base, by using the pairwise comparison of receiver operating characteristics (ROC) curves based on the method of Delong *et al*.^25^.

Concordance rate and diagnostic accuracy of PET/MRI in both concordant and discordant cases were calculated as well. The word concordance was used when the benign and malignant diagnoses were the same between PET and MRI and the word discordance was used when it was not the same.

For two-tiered classification of the certainty of imaging interpretation, the score 2–4 were considered as unconfident diagnosis and the scores 1 and 5 were considered as confident diagnosis. Categorical variables were compared using Pearson’s chi-square test or Fisher’s exact test as appropriate. Data were analyzed with the use of a commercial statistics package (MedCalc, version 12, MedCalc Software, Mariakerke, Belgium; SPSS, version 21, Chicago, IL). In all tests, *p* values less than 0.05 were considered statistically significant.

### Ethical approval

Institutional review board approved this study.

### Informed consent

Informed consent was waived because of retrospective nature of this study.

## Results

### Diagnostic performances

Simultaneous PET/MRI had a sensitivity of 85.7%, a specificity of 89.1%, a PPV of 89.6%, a NPV of 85.1%, and an accuracy of 87.3%.

Table 2 reveals the comparison of areas under the curve (AUCs) among PET, MRI, and PET/MRI.

**Table 2:** Pairwise comparison of the diagnostic performances of each modality based on receiver operating characteristic curve analysis.

Category		Modality	AUC (95% CI)	P-value^†^	P-value^‡^
Per patient (N = 73)		PET	0.747 (0.361-0.841)		0.57
		MRI	0.798 (0.687-0.883)	0.57	
		PET/MRI	0.934 (0.850-0.979)	<0.05	<0.05
Per Lesion (N = 134)		PET	0.847 (0.775-0.904)		0.73
		MRI	0.836 (0.762-0.894)	0.73	
		PET/MRI	0.926 (0.868-0.964)	<0.05	<0.05
	Non-nodal lesion (N=72)	PET	0.726 (0.608-0.825)		<0.05
		MRI	0.859 (0.756-0.929)	<0.05	
		PET/MRI	0.928 (0.842-0.976)	<0.05	0.0722
	Nodal lesion (N = 62)	PET	0.859 (0.756-0.929)		0.0512
		MRI	0.804 (0.683-0.894)	0.0512	
		PET/MRI	0.863 (0.752-0.937)	0.8361	<0.05
	Initial work-up (N = 79)	PET	0.827 (0.726-0.903)		0.6974
		MRI	0.812 (0.709-0.891)	0.6974	
		PET/MRI	0.888 (0.798-0.948)	0.0840	<0.05
	Work-up for recurrence (N = 55)	PET	0.845 (0.722-0.929)		0.6559
		MRI	0.875 (0.758-0.949)	0.6559	
		PET/MRI	0.987 (0.911-1.000)	<0.05	<0.05

Note > AUC = Area under the curve, ^†^P-values from the comparison with PET, ^‡^P-values from the comparison with MRI, The 95% confidence interval values are demonstrated in parenthesis.

In the assessment per patient (n = 73) and per lesion (n = 134), AUC of PET/MRI was higher than both PET and MRI, respectively (P < 0.05). In the assessment of non-nodal lesions (n = 72), AUCs of both PET/MRI and MRI were higher than PET (P < 0.05). In the assessment of nodal lesions (n = 62), AUC of PET/MRI was higher than MRI (P < 0.05). PET/MRI revealed higher AUC than MRI for initial work-up (P < 0.05) and higher AUC than both PET and MRI for work-up of recurrence (P < 0.05, respectively). Whether it was statistically significant or not, AUC of PET/MRI tended to be largest in analyses of all above categories than both PET and MRI.

### Concordant and discordant diagnosis

Combinations of two techniques showed concordance rates of 82.8% (111 /134; benign concordant case, n = 52; malignant concordant case, n = 59) and discordant cases of 17.2% (23/134).

**Figure 1:**
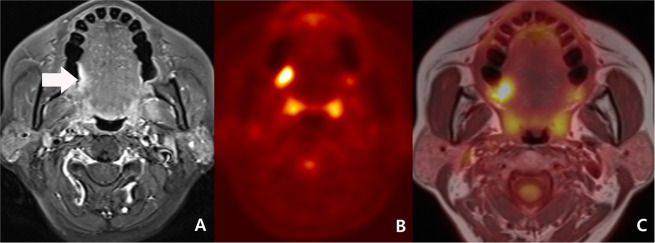
Initial work-up of a 53-year-old woman with right tongue cancer (malignant concordant diagnosis) and bilateral cervical metastasis (benign concordant diagnosis). (**A**) A transverse fat-suppression contrast enhanced MRI revealed an enhancing lesion at right posterolateral tongue (arrow). A neuroradiologist scored the lesion as 4 (probably malignant). (**B**) As PET reveals hypermetabolism (maximal SUV: 9.4) at the corresponding area, a nuclear medicine specialist scored the lesion as 5 (definitely malignant). (**C**) A consensus score of PET/MRI was 5 (definitely malignant). Right tongue lesion was surgically proved to be squamous cell carcinoma. In addition, there were numerous tiny lymph node metastases proven by surgical specimens of right neck dissection, although imaging with PET, MRI, and PET/MRI could not detect them.

In concordant cases, PET/MRI had a sensitivity of 87.3%, a specificity of 91.7%, a PPV of 93.2%, a NPV of 84.6%, and an accuracy of 89.2%. (Fig. 1)

**Figure 2:**
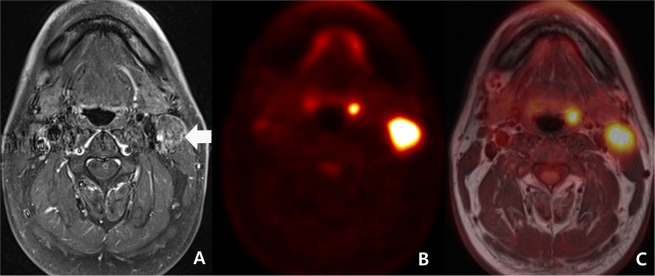
Initial work-up of a 54-year-old man with left palatine tonsillar cancer (discordant diagnosis) and ipsilateral cervical lymph node metastasis (malignant concordant diagnosis). (**A**) A transverse fat-suppression contrast enhanced MRI revealed enhancing lesion at left cervical chain (arrow). A neuroradiologist scored the lesion as 5 (definitely malignant). However, he failed to reveal primary focus in the scan. (**B**) As PET reveals hypermetabolism (maximal SUV: 12.3) at the left cervical chain, a nuclear medicine specialist scored the lesion as 5 (definitely malignant). In addition, because PET shows a small hypermetabolic lesion at left palatine tonsil (maximal SUV: 8.3), he scored the lesion as 4 (probably malignant). (**C**) A consensus score of PET/MRI for left cervical lesion was 5 (definitely malignant) and a consensus score of the left palatine tonsillar lesion was 4 (probably malignant). Both left cervical chain lesion and left palatine tonsillar lesion were proved to be squamous cell carcinoma.

In discordant cases, PET/MRI had a sensitivity of 71.4%, a specificity of 81.3%, a PPV of 62.5%, a NPV of 86.7%, and an accuracy of 78.3%. (Fig. 2)

Independently in these discordant cases, PET had a sensitivity of 42.9%, a specificity of 50.0%, a PPV of 27.3%, a NPV of 66.7%, and an accuracy of 47.8%. MRI had a sensitivity of 57.1%, a specificity of 50.0%, a PPV of 33.3%, a NPV of 72.7%, and an accuracy of 52.2%. The 11 discordant cases with malignant diagnosis on PET and benign diagnosis on MRI comprised 9 non-nodal lesions and 2 nodal lesions. Among them, MRI was correct in 6 out of 9 non-nodal lesions and 2 out of 2 nodal lesions. The 12 discordant cases with benign diagnosis on PET and malignant diagnosis on MRI comprised 4 non-nodal lesions and 8 nodal lesions. Among them, PET was correct in 8 out of 8 nodal lesions and MRI was correct in 4 out of 4 non-nodal lesions.

While NPV was not statistically different between benign concordant cases (84.6%, 44/52) and discordant cases (86.7%, 13/15), PPV was larger in malignant concordant cases (93.2%, 55/59) than in discordant cases (62.5%, 5/8) (p = 0.0404) (Figs. 1, 2).

### Diagnostic confidence of PET/MRI

For all lesions, confident scoring rates were 67.2% (90/134) in MRI, 82.8% (111/134) in PET, and 76.9% (103/134) in PET/MRI, respectively.

In benign concordant cases, confident scoring rates were 75.0% (39/52) for MRI, 82.7% (43/52) for PET, and 80.8% (42/52) for PET/MRI, respectively.

In malignant concordant cases, confident scoring rate of PET/MRI (96.6% [57/59]) tended to be larger than MRI (76.3% [45/59], p = 0.0031) and PET (89.8% [53/59], p = 0.2720), respectively (Fig. 1). In discordant cases, confident scoring rate of PET/MRI (17.4% [4/23])

tended to be lower in MRI (26.1% [6/23], p = 0.7207) and PET (65.2% [15/23], p = 0.0027), respectively.

## Discussion

Our study first presented better diagnostic performance of PET/MRI in comparison with PET or MRI alone for diagnosing malignant lesions in patients with head and neck cancer, for both per lesion base and per patient base. In addition, the tendency toward better diagnostic performance of PET/MRI was also presented in various clinical settings as follows; both initial and follow-up work-ups and both nodal and non-nodal lesions.

The result of our PET/MRI study might be in line with the result of previous studies where PET/CT was shown to be more accurate than PET or CT alone^4,5^.

Furthermore, in published literature, PET/MRI has been reported to be generally equivalent and sometimes better diagnostic potential when compared to PET/CT in the head and neck region^2,10,14,15,26,27^.

For the lesion evaluation in head and neck region, CT has the advantages of relatively easy accessibility and less motion artifact. However, additional MRI may be needed even in the case where PET/CT has already been obtained. This is because MRI has a definite advantage of better soft tissue contrast in complex anatomical structure and depiction of the tumor involving bone marrow in craniofacial bones in addition to the intrinsic advantage of no radiation and less metallic artifact from dental prostheses^10,13,14,28–32^.

Meanwhile, this study first presented that more than 80% of cases were diagnostically concordant between PET and MRI. However, irrespective of concordant tendency, this study showed another advantage of PET/MRI with higher PPV in malignant concordant lesions than in discordant lesions. Also, PET/MRI enhanced diagnostic confidence when compared to PET and MRI alone in malignant concordant cases.

**Figure 3:**
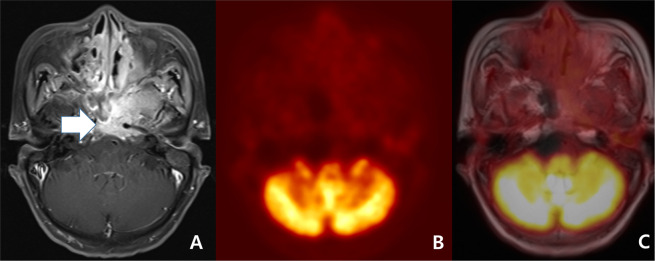
Follow-up work-up of a 69-year-old woman with nasopharyngeal carcinoma treated by concurrent chemoradiation therapy. (**A**) A transverse fat-suppression contrast enhanced MRI revealed enhancing lesion at nasopharynx and mid skull base (arrow). The lesion decreased in size when compared to pre-treatment MRI (not displayed). However, a neuroradiologist scored the lesion as 5 (definitely malignant). (**B**) The PET reveals equivocally mild metabolism (maximal SUV: 2.9). Considering the interval significant decrease of the metabolism (maximal SUV on pre-treatment PET: 9.3 [not displayed]), a nuclear medicine specialist scored the lesion as 2 (probably benign). (**C**) A consensus score of PET/MRI for the lesion at nasopharynx and mid skull base was 4 (probably malignant) and biopsy specimen revealed a residual tumor.

However, in spite of these advantages, we must be aware that PET/MRI also has various pitfalls. First, in even benign concordant cases, PET/MRI might have produced false negative results in cases such as small lesions (Fig. 1)^10,20^. Second, in even malignant concordant cases, PET/MRI might have produced false positive results in cases such as inflammatory lymph node or lymphoid hyperplasia that can be seen in a larger size on the MRI and as hypermetabolism on PET^3,9,11,20^. Third, in discordant cases, MRI has been known to show better diagnostic accuracy in necrotic tumor, perineural spread, and tumor with low metabolic activity such as low grade lymphoma (Fig. 3)^4,10,14,26,33–35^. In contrast, PET might have better advantage in small but FDG-avid tumor and work-up for metastasis of unknown origin (Fig. 2)^9,11,36^. In the discordant cases of our study, PET tended to be correct in nodal lesions but MRI tended to be correct in non-nodal lesions.

Retrospective fusion between PET and MRI might produce similar diagnostic accuracy of simultaneous PET/MRI^9^. However, simultaneous PET/MRI may result in a better alignment of both imaging data because it eliminates patient movements during repositioning^12,33^. In addition, it gives the patients a unique advantage with reduced time and lowered medical cost by offering information which simultaneously combines PET and MRI, both of which may be essential for the evaluation of head and neck lesions. Furthermore, this simultaneous system will play a greater role in future multiparametric comparative analysis that combines multiple functional information from various advanced MRI techniques (e.g. diffusion-weighted image, perfusion-weighted image, MR spectroscopy, etc.) and metabolic information from PET^11,15,37–39^. Although this study has an intrinsic limitation of being a retrospective study with a small number of cases, it succeeded in classifying the diagnostic usefulness in accuracy and consistency. Further larger prospective study including the role of multiparametric analysis is necessary to validate the usefulness of PET/MRI.

In conclusion, PET/MRI shows better diagnostic performance when compared with PET or MRI alone for depicting malignant lesions in the head and neck.
